# AteMeVs: An R package for the estimation of the average treatment effect with measurement error and variable selection for confounders

**DOI:** 10.1371/journal.pone.0296951

**Published:** 2024-09-30

**Authors:** Li-Pang Chen, Grace Y. Yi

**Affiliations:** 1 Department of Statistics, National Chengchi University, Taipei, Taiwan, ROC; 2 Department of Statistical and Actuarial Sciences, Department of Computer Science, University of Western Ontario, London, Canada; The First Hospital of Jilin University, CHINA

## Abstract

In causal inference, the estimation of the average treatment effect is often of interest. For example, in cancer research, an interesting question is to assess the effects of the chemotherapy treatment on cancer, with the information of gene expressions taken into account. Two crucial challenges in this analysis involve addressing measurement error in gene expressions and handling noninformative gene expressions. While analytical methods have been developed to address those challenges, no user-friendly computational software packages seem to be available to implement those methods. To close this gap, we develop an R package, called **AteMeVs**, to estimate the average treatment effect using the inverse-probability-weighting estimation method to handle data with both measurement error and spurious variables. This developed package accommodates the method proposed by Yi and Chen (2023) as a special case, and further extends its application to a broader scope. The usage of the developed R package is illustrated by applying it to analyze a cancer dataset with information of gene expressions.

## 1 Introduction

Bioinformatics has revealed that cancer stems from a genetic disorder, deiven by genetic variations that lead to the abnormally dysfunction of genes and their altered expressions (e.g., [1]). Accurate assessment of gene expression levels becomes crucial for cancer diagnosis and treatment. Chemotherapy is a commonly used approach in cancer treatment as it often effectively eradicates malignant cells. In particular, the integration of targeted therapy with chemotherapy is frequently used to control the growth, division, and spread of cancer cells (e.g., [2]). However, due to its lack of specificity in targeting cancer cells, chemotherapy drugs can impact both cancer cells and healthy cells, which leads to significant side effects. One concern is whether employing chemotherapy is more beneficial than taking alternative treatments that avoid it. We are interested in studying whether taking chemotherapy has a causal effect on increasing the survival of cancer patients.

**Fig 1:**
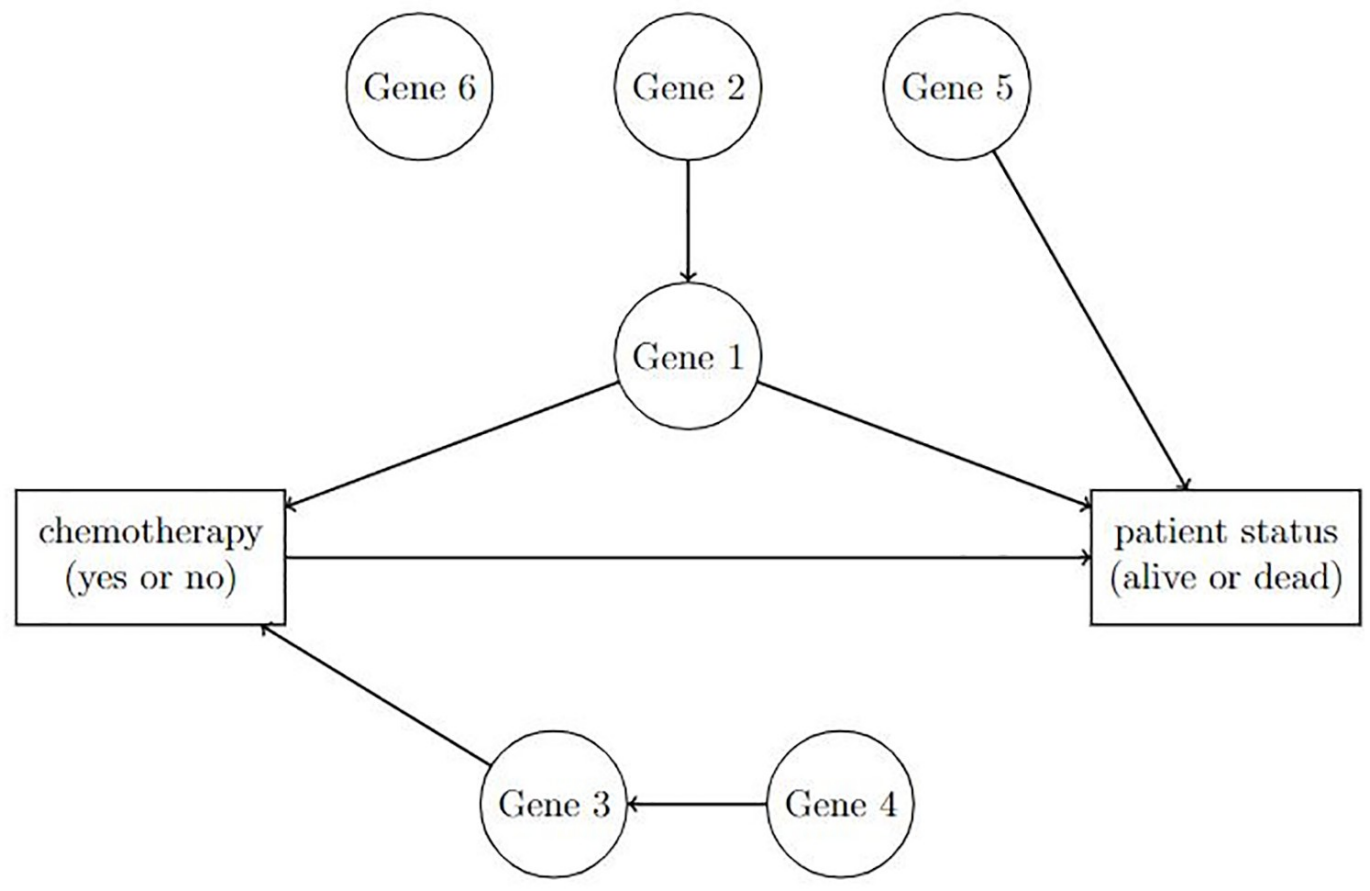
An illustrative diagram of the causal relationship with possible confounders.

This research is partially motivated by the Molecular Taxonomy of Breast Cancer International Consortium (METABRIC) database, a Canada-UK Project that includes targeted sequencing data of primary breast cancer samples collected by the Cambridge Research Institute and the British Columbia Cancer Centre in Canada [3]. The dataset with all gene expression names is publicly available on the Kaggle website (https://www.kaggle.com/datasets/raghadalharbi/breast-cancer-gene-expression-profiles-metabric). One interesting question is whether patients taking chemotherapy as a treatment (“hormone_therapy”: 1 is yes and 0 is no) can increase the chance of the survival status (“overall_survival”: 1 is alive and 0 is dead). The dataset contains Z-scores of m-RNA levels for 331 genes, where the Z-score is defined as

$$\begin{array}{c} \frac{\text{an}\;\text{expression}\;\text{level}\;\text{in}\;\text{the}\;\text{tumor}\;\text{sample}\;-\;\text{the}\;\text{mean}\;\text{expression}\;\text{in}\;\text{reference}\;\text{sample}}{\text{standard}\;\text{deviation}\;\text{of}\;\text{expression}\;\text{levels}\;\text{in}\;\text{reference}\;\text{sample}}, \end{array}$$

which is a continuous variable. In addition, some gene expressions may be confounded with the outcome and treatment, shown in Fig 1.

Taking the causal inference paradigm, we formulate the question as the estimation of the average treatment effect (ATE), defined as the difference between potential outcomes under two treatments, where the two treatments refer to taking and not taking chemotheropy, respectively.

Various causal inference methods, accompanied by R packages, are available in the literature. Examples include **iWeigReg** [4], **SVMMatch** [5], **CausalGAM** [6], and **wfe** [7]. The R package **mediation** [8] is developed to conduct mediation analysis. Package **qualCI** [9] is used to analyze causal inference with qualitative and ordinal information on outcomes. **MatchingFrontier** [10] applies the matching method to handle the balance issue in causal inference.

In the framework of causal inference, the inverse probability weighted (IPW) estimation method has been widely used to estimate average treatment effects due to its simplicity and transparent interpretation (e.g., [11–13]). The method adjusts for the effects of measured confounders by re-weighting the data as if the weighted data were collected from randomized controlled trials. The validity of the method requires two key conditions: (1) the treatment model is correctly specified to consistently estimate propensity scores, and (2) the variables in the treatment model are precisely measured.

In the presence of measurement error, directly applying those methods to the observed data usually yields unreliable estimation results. As illustrated in Fig 1, in the study of the treatment effect on the outcome, confounders (e.g., gene expressions) may possess complex features: some are associated only with the treatment variable, some are solely related to the outcome variable, some are connected to both treatment and outcome variables, and some are not relevant to either treatment or outcome variable. Including irrelevant confounders in the analysis or model building may lead to misleading results.

In the literature, variable selection for causal inference (e.g., [14–16]) or measurement error correction (e.g., [17–22]) are discussed under various settings. However, in the concurrent presence of both features, limited work has been carried out to estimate ATE except for [23]. Moreover, there is a lack of user-friendly R packages designed to facilitate causal estimation for data with both measurement error and spurious variables.

In this paper, we develop an R package, called **AteMeVs**, which is desired to estimate the average treatment effect with measurement error and variable selection for confounders. This package, available at CRAN [24], is developed to implement a recent method proposed by [23], which estimates the average treatment effect for noisy data that include both measurement error and spurious variables needed to be excluded. The developed package contains a set of functions that provide a step-by-step estimation procedure, including the correction of the measurement error effects, variable selection for the estimation of propensity scores, and estimation of ATE. Our functions contain multiple options for users to implement, including different ways to correct for the measurement error effects, various penalty functions for variable selection, and different regression models for characterizing propensity scores.

## 2 Notation and framework

### 2.1 Propensity score

In contrast to the variables indicated by Fig 1, we now introduce abstract symbols to classify the associated variables differently. Let *T* denote the observed binary treatment (e.g., chemotherapy) with *T* = 1 if treated and *T* = 0 if untreated. Accordingly, we consider counterfactual responses corresponding to the treatment status. For *t* ∈ {0, 1}, let *Y*_(*t*)_ represent the potential outcome of the patient if the patient would have received *T* = *t*. As described in Section 1, the goal is to assess the causality effect of the treatment (e.g., chemotherapy) on increasing the patient’s survival, or equivalently, we are interested in estimating the ATE,

$$\begin{array}{c} \tau_{0}≜E\left(Y_{\left(1\right)}\right)-E\left(Y_{\left(0\right)}\right). \end{array}$$

To facilitate an individual’s characteristics, we let *W* denote the *p*-dimensional vector of pre-treatment confounders for the individual, which as an example, can be understood as gene expressions in Fig 1. To reflect the possible dependence of *T* on *W*, we consider the conditional probability

$$\begin{array}{c} \pi ≜P(T=1|W), \end{array}$$

also called the propensity score for the individual. The introduction of the propensity score allows us to use the observed outcome, denoted by *Y*, and the treatment information to consistently estimate *τ*_0_ (e.g., [25]), which basically is due to the property (e.g., [23])

$$\begin{array}{c} \tau_{0}=E(\frac{TY}{\pi })-E\{\frac{(1-T)Y}{1-\pi }\}. \end{array}$$ (1)

The validity of (1) hinges on the following standard assumptions in the causal inference framework:

1. The strong ignorable treatment assumption (SITA): given the covariates *W*, potential outcomes *Y*_(0)_ and *Y*_(1)_ are independent of *T*;
2. The stable unit treatment value assumption (SUTVA), also known as the consistency assumption: each subject’s potential outcomes are not influenced by the actual treatment assignment of other subjects. Therefore, the observed outcome, *Y*, for an individual is assumed to be linked with potential outcomes via *Y* = *TY*_(1)_ + (1 − *T*)*Y*_(0)_;
3. The positivity assumption: the propensity score is between 0 and 1, i.e., 0 < *π*(*W*) < 1 for all *W*.

In applications, *π* is frequently characterized by a parametric model:

$$\begin{array}{c} g^{-1}\left(\pi \right)=W^{⊤}\gamma , \end{array}$$ (2)

where *γ* = (*γ*_0_, *γ*_1_, ⋯, *γ*_*p*_)^⊤^ is the vector of regression parameters of dimension *p* + 1, with *γ*_0_ representing the intercept; and *g*(⋅) is a link function. For ease of exposition and the inclusion of the intercept, we slightly abuse the notation *W* in (2) by including 1 to the original *p*-dimensional vector of confounders here and in the subsequent development. Common choices of *g*(⋅) include the logit, probit, and complementary log-log functions, respectively yielding

- *the logistic regression model*:
  $$\begin{array}{c} \pi =\frac{\text{exp}\;(W^{⊤}\gamma )}{1+\text{exp}\;(W^{⊤}\gamma )}, \end{array}$$ (3)
- *the probit regression model*:
  $$\begin{array}{c} \pi =\Phi (W^{⊤}\gamma ), \end{array}$$ (4)
  with Φ(⋅) representing the cumulative distribution function of the standard normal distribution, and
- *the complementary log-log regression model*:
  $$\begin{array}{c} \pi =1-\text{exp}\;\{-\text{exp}\;\left(W^{⊤}\gamma \right)\}. \end{array}$$ (5)

### 2.2 The IPW estimator

With the setup in Section 2.1, the estimation of *τ*_0_ can be carried out using the measurements of a random sample, say {{*T*_*i*_, *Y*_*i*_, *W*_*i*_}: *i* = 1, …, *n*} of size *n*, where *T*_*i*_, *Y*_*i*_, and *W*_*i*_ represent the corresponding variables for subject *i* with *i* = 1, …, *n*.

The estimation of *τ*_0_ basically involves the following two steps. In the first step, we estimate the propensity score *π*_*i*_ = *P*(*T*_*i*_ = 1|*W*_*i*_) for subject *i* = 1, …, *n* based on estimating parameter *γ* in model (2). Let *S*_*i*_(*γ*;*W*_*i*_) denote the likelihood score function obtained from subject *i* that is derived from fitting model (2). If the true value of *W*_*i*_ is available, one may solve

$$\begin{array}{c} \sum_{i=1}^{n}S_{i}\left(\gamma ;W_{i}\right)=0 \end{array}$$ (6)

for *γ* to obtain a consistent estimate of *γ*, denoted $\hat{\gamma }$, provided usual regularity conditions. Then we calculate *π*_*i*_ with *γ* in (2) replaced by the estimate $\hat{\gamma }$, and let ${\hat{\pi }}_{i}$ denote the resulting value of *π*_*i*_.

In the second step, utilizing the property (1) yields a consistent estimate of the ATE *τ*_0_ by the following IPW estimator, as initiated by [25]:

$$\begin{array}{c} \hat{\tau }=\frac{1}{n}\sum_{i=1}^{n}\frac{T_{i}Y_{i}}{{\hat{\pi }}_{i}}-\frac{1}{n}\sum_{i=1}^{n}\frac{\left(1-T_{i}\right)Y_{i}}{1-{\hat{\pi }}_{i}}. \end{array}$$ (7)

To mitigate unstable numerical results caused by extreme values of ${\hat{\pi }}_{i}$ that may be close to 0 or 1, [12] proposed a stable version of (7), which also offers a consistent estimator of *τ*_0_:

$$\begin{array}{c} \hat{\tau }={\left(\sum_{i=1}^{n}\frac{T_{i}}{{\hat{\pi }}_{i}}\right)}^{-1}\sum_{i=1}^{n}\frac{T_{i}Y_{i}}{{\hat{\pi }}_{i}}-{\left(\sum_{i=1}^{n}\frac{1-T_{i}}{1-{\hat{\pi }}_{i}}\right)}^{-1}\sum_{i=1}^{n}\frac{\left(1-T_{i}\right)Y_{i}}{1-{\hat{\pi }}_{i}}. \end{array}$$ (8)

We use (8) for the following development.

### 2.3 Irrelevant variables and measurement error

As noted in [23], the validity of (7) or (8) breaks down in the presence of two features of noisy data: measurement error and irrelevant variables.

In applications, some variables (e.g., gene expressions) in *W*_*i*_ can be subject to measurement error. To reflect this feature, we write *W*_*i*_ as ${\left(X_{i}^{⊤},Z_{i}^{⊤}\right)}^{⊤}$ so that all error-prone variables are included in *X*_*i*_ and all precisely measured variables go to *Z*_*i*_. Let $X_{i}^{*}$ denote the observed surrogate measurement of *X*_*i*_.

To characterize the relationship between $X_{i}^{*}$ and *X*_*i*_, we consider the classical additive error model (e.g., [26, 27])

$$\begin{array}{c} X_{i}^{*}=X_{i}+e_{i}, \end{array}$$ (9)

where the error term *e*_*i*_ is independent of {*T*_*i*_, *X*_*i*_, *Z*_*i*_, *Y*_*i*_} and follows *N*(0, Σ_*e*_) with covariance matrix Σ_*e*_.

Model (9) is the most commonly used in the literature; it facilitates situations where the observed value fluctuates around the true value with an error term, and the degree of measurement error in $X_{i}^{*}$ is reflected by the value of Σ_*e*_. In this paper, we consider the following four cases for Σ_*e*_:

**Case 1**: Σ_*e*_ is known;
**Case 2**: Σ_*e*_ is unknown and estimated from repeated surrogate measurements $\left\{X_{ij}^{*}:j=1,\cdots ,n_{i};i\in R\right\}$ for a subset, say R, of {1, 2, ⋯, *n*} with |R|=m and *m* < *n*;
**Case 3**: Σ_*e*_ is unknown and estimated from repeated surrogate measurements $\left\{X_{ij}^{*}:j=1,\ldots ,n_{i}\right\}$ of *X*_*i*_ for *i* = 1, ⋯, *n*, where *n*_*i*_ ≥ 2 that may or may not depend on *i*;
**Case 4**: Σ_*e*_ is unknown and estimated from an external validation sample $\left\{\left\{X_{k},X_{k}^{*}\right\}:k\in V\right\}$, where V is index set for the subjects in the validation sample.

Case 1 is useful for addressing data issues in which the existence of measurement error is acknowledged, yet the magnitude of this error has not been quantified. In this case, we often conduct sensitivity analyses to assess the sensitivity of inference results to varying degrees of measurement error, where user-specified values for Σ_*e*_ are typically used to describe different scenarios of measurement error in *X*_*i*_ (e.g., [27, 28]). This case was also considered by [23]. Cases 2 and 3 complement each other in addressing two scenarios with repeated surrogate measurements. In contrast to the availability of replicates, Case 4 assumes the availability of an external validation dataset.

The second issue of noisy data concerns irrelevant variables in the data. As shown in Fig 1, some gene expressions have no connection with chemotherapy or patient’s survival status. To reflect this feature, we write $W_{i}={\left(W_{\text{I}i}^{⊤},W_{\text{II}i}^{⊤}\right)}^{⊤}$ for *i* = 1, ⋯, *n*, where $W_{\text{I}i}$ includes the informative confounders associated with *T*_*i*_ and *Y*_*i*_, and $W_{\text{II}i}$ contains noninformative confounders. We further write $W_{\text{I}i}={\left(Z_{\text{I}i}^{⊤},X_{\text{I}i}^{⊤}\right)}^{⊤}$ and $W_{\text{II}i}={\left(Z_{\text{II}i}^{⊤},X_{\text{II}i}^{⊤}\right)}^{⊤}$ so that $Z_{i}≜{\left(Z_{\text{I}i}^{⊤},Z_{\text{II}i}^{⊤}\right)}^{⊤}$ represents the subvector of error-free confounders in *W*_*i*_ and $X_{i}≜{\left(X_{\text{I}i}^{⊤},X_{\text{II}i}^{⊤}\right)}^{⊤}$ is the subvector of error-prone confounders in *W*_*i*_. Let $p_{Z}$ and $p_{X}$ denote the dimension of *Z*_*i*_ and *X*_*i*_, respectively. We let $X_{i}^{*}={\left(X_{\text{I}i}^{*⊤},X_{\text{II}i}^{*⊤}\right)}^{⊤}$ denote the observed version of *X*_*i*_, where $X_{\text{I}i}^{*}$ and $X_{\text{II}i}^{*}$ are the observed measurements of $X_{\text{I}i}$ and $X_{\text{II}i}$, respectively.

## 3 Estimation methods

Here we describe methods for estimating the average treatment effect *τ*_0_, with the features of variable selection and measurement error accommodated for each of the four cases described in Section 2.3. The main idea comes from Section 3.1 of [23], which is developed under Case 1 described in Section 2.3. Sections 3.2-3.4 extend the development in Section 3.1 to respectively handle Cases 3.2-3.4 described in Section 2.3.

### 3.1 Implementation steps for Case 1

First, we describe the algorithm for Case 1 where Σ_*e*_ is user-specified. The algorithm of estimating *τ*_0_ contains the five steps, summarized as follows. For details, see Section 3.1 of [23].

**Step 1. Simulation**:

We simulate a sequence of artificial surrogates, denoted $\left\{X_{i}^{*}\left(k,\psi \right):k=1,\cdots ,K;\psi \in C;i=1,\cdots ,n\right\}$, where *K* is a user-specified positive integer, $C=\{\psi_{1},\psi_{2},\ldots ,\psi_{\text{M}}\}$ is a sequence of *M* non-negative values taken from $\left[0,\psi_{\text{M}}\right]$ with a given $\psi_{\text{M}}$ and *ψ*_1_ = 0, and

$$\begin{array}{c} X_{i}^{*}\left(k,\psi \right)=X_{i}^{*}+\sqrt{\psi }e_{ik}\;\text{with}\;e_{ik}\;\text{independently}\;\text{generate}\;\text{from}\;N\left(0,\Sigma_{e}\right). \end{array}$$ (10)

**Step 2. Estimation of Treatment Model Parameters**:

Parameter *γ* in model (2) is estimated by solving (6) with *X*_*i*_ replaced by $X_{i}^{*}\left(k,\psi \right)$, and let $\hat{\gamma }\left(k,\psi \right)$ denote the resulting estimate. Calculate $\hat{\gamma }\left(\psi \right)=K^{-1}\sum_{k=1}^{K}\hat{\gamma }\left(k,\psi \right)$ for ψ∈C.

**Step 3. Extrapolation**:

For *j* = 0, 1, 2, …, *p*, let ${\hat{\gamma }}_{j}\left(\psi \right)$ denote the *j*th element of $\hat{\gamma }\left(\psi \right)$; fit a regression model to $\left\{\left(\psi ,{\hat{\gamma }}_{j}\left(\psi \right)\right):\psi \in C\right\}$ and extrapolate it to *ψ* = −1; and let ${\tilde{\gamma }}_{j}$ denote the resulting extrapolated value of *γ*_*j*_, the *j*th element of *γ*. Write $\tilde{\gamma }={\left({\tilde{\gamma }}_{0},{\tilde{\gamma }}_{1},\ldots ,{\tilde{\gamma }}_{p}\right)}^{⊤}$.

**Step 4. Variable Selection**:

Minimize the penalized quadratic loss function

$$\begin{array}{ccc} \ell_{\text{P}}\left(\gamma \right) & ≜ & \ell \left(\gamma \right)-n\sum_{j=1}^{p}\rho_{\lambda }\left(\right|\gamma_{j}\left|\right)\\ & ≜ & \frac{1}{2}{\left(\gamma -\tilde{\gamma }\right)}^{⊤}V_{n}\left(\gamma -\tilde{\gamma }\right)-n\sum_{j=1}^{p}\rho_{\lambda }\left(\right|\gamma_{j}\left|\right) \end{array}$$ (11)

with respect to *γ*, where *ρ*_λ_(⋅) is a user-specified penalty function with a tuning parameter λ, and *V*_*n*_ is a user-specified positive definite weight matrix.

**Step 5. Estimation of ATE**:

Write $\hat{\gamma }={\left({\hat{\gamma }}_{\text{I}}^{⊤},{\hat{\gamma }}_{\text{II}}^{⊤}\right)}^{⊤}$ with ${\hat{\gamma }}_{\text{I}}={\left({\hat{\gamma }}_{x\text{I}}^{⊤},{\hat{\gamma }}_{z\text{I}}^{⊤}\right)}^{⊤}$ and ${\hat{\gamma }}_{\text{II}}={\left({\hat{\gamma }}_{x\text{II}}^{⊤},{\hat{\gamma }}_{z\text{II}}^{⊤}\right)}^{⊤}$ corresponding to the non-zero and zero components in $\hat{\gamma }$, respectively. With unimportant variables $X_{\text{II}i}$ and $Z_{\text{II}i}$ excluded from the initial model (2), the final treatment model is taken as

$$\begin{array}{c} g^{-1}\left(\pi_{i}\right)=W_{\text{I}i}^{⊤}\gamma_{\text{I}}, \end{array}$$ (12)

where $\gamma_{\text{I}}$ is the vector of model parameters associated with important covariates $W_{\text{I}i}$.

For *k* = 1, ⋯, *K* and ψ∈C, calculate an estimate, say, $\hat{\tau }\left(k,\psi \right)$, of *τ*_0_ using (8) with ${\hat{\pi }}_{i}$ replaced by the propensity score for subject *i*, determined by the selected treatment model (12) with $X_{\text{I}i}$ replaced by $X_{\text{I}i}^{*}\left(k,\psi \right)$, the subvector of $X_{i}^{*}\left(k,\psi \right)$ that corresponds to $X_{\text{I}i}^{*}$. Then calculate

$$\hat{\tau }\left(\psi \right)=K^{-1}\sum_{k=1}^{K}\hat{\tau }\left(k,\psi \right).$$

Finally, fit a regression model to $\left\{\left(\psi ,\hat{\tau }\left(\psi \right)\right):\psi \in C\right\}$ and extrapolate it to *ψ* = −1. The resulting value, denoted as $\hat{\tau }$, is taken an estimate of *τ*_0_.

### 3.2 Implementation steps for Case 2

Consider Case 2 where repeated measurements $\left\{X_{ij}^{*}:j=1,\cdots ,n_{i};i\in R\right\}$ of *X*_*i*_ are available for |R|≜m subjects, and surrogates $X_{ij}^{*}$ and *X*_*i*_ are linked via the measurement error model

$$\begin{array}{c} X_{ij}^{*}=X_{i}+e_{ij}\;\;\text{for}\;\;i\in R\;\;\text{and}\;\;j=1,\cdots ,n_{i}, \end{array}$$

where for $i\in R,n_{i}\geq 2$; *e*_*ij*_ follows *N*(0, Σ_*e*_) with unknown covariance matrix Σ_*e*_; and the *e*_*ij*_ are independent of {*T*_*i*_, *X*_*i*_, *Z*_*i*_, *Y*_*i*(1)_, *Y*_*i*(0)_}.

With the repeated measurements, using the method of moments, we estimate Σ_*e*_ by

$$\begin{array}{c} {\hat{\Sigma }}_{e}=\frac{\sum_{i\in R}\sum_{j=1}^{n_{i}}(X_{ij}^{*}-{\bar{X}}_{i}^{*}){\left(X_{ij}^{*}-{\bar{X}}_{i}^{*}\right)}^{⊤}}{\sum_{i\in R}(n_{i}-1)}, \end{array}$$ (13)

where ${\bar{X}}_{i}^{*}=n^{-1}\sum_{j=1}^{n_{i}}X_{ij}^{*}$.

To estimate *τ*_0_, we repeat the five steps described in Section 3.1 with Σ_*e*_ in (10) replaced by (13).

### 3.3 Implementation steps for Case 3

Now we consider Case 3 described in Section 2.3, where Σ_*e*_ is unknown but repeated surrogate measurements $\left\{X_{ij}^{*}:j=1,\cdots ,n_{i}\right\}$ are available for all the subjects in the sample, with *n*_*i*_ ≥ 2 for *i* = 1, ⋯, *n*.

Adapting the development in [27, 29] (p.107), we modify Step 1 in Section 3.1 as follows. For any ψ∈C and *i* = 1, ⋯, *n*, we generate *n*_*i*_ variates independently from the standard normal distribution, and let {*d*_*ij*_(*ψ*): *j* = 1, ⋯, *n*_*i*_} denote them. Calculate ${\bar{d}}_{i}\left(\psi \right)=\frac{1}{n_{i}}\sum_{j=1}^{n_{i}}d_{ij}\left(\psi \right)$ and

$$\begin{array}{c} c_{ij}\left(\psi \right)=\frac{d_{ij}\left(\psi \right)-{\bar{d}}_{i}\left(\psi \right)}{\sqrt{\sum_{l=1}^{n_{i}}{\left\{d_{il}\left(\psi \right)-{\bar{d}}_{i}\left(\psi \right)\right\}}^{2}}}. \end{array}$$

Then for *k* = 1, ⋯, *K*, we define

$$\begin{array}{c} X_{i}^{**}\left(k,\psi \right)={\bar{X}}_{i}^{*}+\sqrt{\frac{\psi }{n_{i}}}\sum_{j=1}^{n_{i}}c_{ij}\left(\psi \right)X_{ij}^{*}, \end{array}$$ (14)

where ${\bar{X}}_{i}^{*}=n_{i}^{-1}\sum_{j=1}^{n_{i}}X_{ij}^{*}$. Estimation of *τ*_0_ can then be proceeded following the five steps in Section 3.1, with $X_{i}^{*}\left(k,\psi \right)$ in (10) replaced by $X_{i}^{**}\left(k,\psi \right)$ in (14).

### 3.4 Implementation steps for Case 4

We now consider Case 4 described in Section 2.3. In this case, we have the main study data, given by $\left\{\{Y_{i},T_{i},Z_{i},X_{i}^{*}\}:i\in M\right\}$ with M={1,⋯,n}, and an external validation sample $\left\{\left\{X_{k},X_{k}^{*}\right\}:k\in V\right\}$ with size |V|≜m, where the index sets M and V do not overlap. We assume that $X_{k}^{*}$ and *X*_*k*_ are related via (9) for k∈V. Further, we make the transportability assumption, considered in [30].

With the availability of *X*_*k*_ and $X_{k}^{*}$ in V, we can empirically estimate Σ_*X*_ = var(*X*_*k*_) and $\Sigma_{X^{*}}=\text{var}\left(X_{k}^{*}\right)$, and denote the resulting estimators by

$$\begin{array}{c} {\hat{\Sigma }}_{X}=\frac{1}{\left|V\right|}\sum_{i\in V}(X_{i}-{\bar{X}}_{i}){\left(X_{i}-{\bar{X}}_{i}\right)}^{⊤} \end{array}$$

and

$$\begin{array}{c} {\hat{\Sigma }}_{X^{*}}=\frac{1}{\left|V\right|}\sum_{i\in V}(X_{i}^{*}-{\bar{X}}_{i}^{*}){\left(X_{i}^{*}-{\bar{X}}_{i}^{*}\right)}^{⊤}, \end{array}$$

respectively, where ${\bar{X}}_{i}=\frac{1}{\left|V\right|}\sum_{i\in V}X_{i}$ and ${\bar{X}}_{i}^{*}=\frac{1}{\left|V\right|}\sum_{i\in V}X_{i}^{*}$.

Consequently, by the additivity of covariance matrices in (9), we estimate Σ_*e*_ by

$$\begin{array}{c} {\hat{\Sigma }}_{e}={\hat{\Sigma }}_{X^{*}}-{\hat{\Sigma }}_{X}. \end{array}$$ (15)

Then estimation of *τ*_0_ is carried out following the five steps in Section 3.1, with Σ_*e*_ in (10) replaced by the estimator ${\hat{\Sigma }}_{e}$ in (15).

## 4 Implementation details

To implement the estimation procedures described in Section 3, we need to first decide the choice of relevant quantities, including *K* and C in Step 1, the regression model for extrapolation in Steps 3 and 5, and the penalty function together with the tuning parameter in Step 4. In the following subsections, we discuss the choice of each quantity individually.

### 4.1 Choice of *K* and C in step 1

Integer *K* determines the repetition of simulated data $X_{i}^{*}\left(k,\psi \right)$ for each given *ψ*. To reduce Monte Carlo errors, a larger value of *K* is expected to produce a more stable result of $\hat{\gamma }\left(\psi \right)$. On the other hand, a larger value of *K* requires a substantially longer computational time. Therefore, a suitable choice of *K* is driven by the trade-off between the computation time and the accuracy of the results. Empirical experience (e.g., [23, 31–33]) suggests setting *K* to be 50, 100, 200, or 500 may be reasonable for many applications.

Regarding the choice of C, one may take $\psi_{\text{M}}$ to be 1 or 2, and divide the interval $\left[0,\psi_{\text{M}}\right]$ equally into *M* sub-intervals, where *M* may be taken as 5, 10, or other positive integers. Then C is the set of the resulting cut points.

### 4.2 Choice of extrapolation function in steps 3 and 5

[26] (Section 5.3.2) suggests to use one of the following functions, denoted by *φ*(*u*) with parameters *β*_0_, *β*_1_, *β*_2_, and *β*_3_, to approximate the true extrapolation functions in implementing Steps 3 and 5:

1. the quadratic function
  $$\begin{array}{c} \varphi \left(u\right)=\beta_{0}+\beta_{1}u+\beta_{2}u^{2}; \end{array}$$ (16)
2. the linear function
  $$\begin{array}{c} \varphi \left(u\right)=\beta_{0}+\beta_{1}u; \end{array}$$ (17)
3. the rational linear function
  $$\begin{array}{c} \varphi \left(u\right)=\beta_{0}+\frac{\beta_{1}}{\beta_{2}+u}. \end{array}$$ (18)
  To increase flexibility, we add the following function to approximate the extrapolation functions for the implementation of Steps 3 and 5:
4. the cubic function
  $$\begin{array}{c} \varphi \left(u\right)=\beta_{0}+\beta_{1}u+\beta_{2}u^{2}+\beta_{3}u^{3}. \end{array}$$ (19)

### 4.3 Choices of penalty function in step 4

In implementing Step 4 in Section 3.1, we consider the following commonly used penalty functions *ρ*_λ_(*u*) that are included in the R package ncvreg:

1. the least absolute shrinkage and selection operator (LASSO) penalty [34]:
  $$\begin{array}{c} \rho_{\lambda }\left(u\right)=\lambda \left|u\right|, \end{array}$$ (20)
2. the smoothly clipped absolute deviation (SCAD) penalty [35]:
  $$\begin{array}{c} \rho_{\lambda }^{\prime }\left(u\right)=\lambda \left\{I\left(u\leq \lambda \right)+\frac{{\left(a\lambda -u\right)}_{+}}{(a-1)\lambda }\cdot I\left(u>\lambda \right)\right\}, \end{array}$$ (21)
  where *I*(⋅) is the indicator function, *u*_+_ = max{*u*, 0}, and *a* = 3.7. Here $\rho_{\lambda }^{\prime }\left(u\right)$ is the first order derivative of the penalty function *ρ*_λ_(*u*) with tuning parameter λ.
3. the minimax concave penalty (MCP) function proposed by [36]:
  $$\begin{array}{c} \rho_{\lambda }^{\prime }\left(u\right)={\left(\lambda -u/a\right)}_{+} \end{array}$$ (22)
  with *a* = 3.
4. the Elastic Net [37]:
  $$\begin{array}{c} \rho_{\lambda }\left(u\right)=\lambda \{\left(1-\alpha \right)u^{2}+\alpha \left|u\right|\}, \end{array}$$ (23)
  with *α* ∈ [0, 1]. If *α* = 1, then (23) reduces to (20); when *α* = 0, then (23) gives the *L*_2_-norma penalty for the ridge regression.

### 4.4 Determination of tuning parameter

To achieve satisfactory performance of the selection procedure, we may consider one of the following criteria for choosing a suitable value for the tuning parameter λ:

1. Bayesian Information Criterion (BIC)
  Given a grid Λ of possible values for the tuning parameter λ, and for λ ∈ Λ, let
  $$\begin{array}{c} \hat{\gamma }\left(\lambda \right)={\text{argmin}}_{\gamma }\ell_{\text{P}}\left(\gamma \right) \end{array}$$
  and let *df*_λ_ denote the number of non-zero elements of $\hat{\gamma }\left(\lambda \right)$. We define
  $$\begin{array}{c} BIC\left(\lambda \right)=-2\ell \left(\hat{\gamma }\left(\lambda \right)\right)+2\left(\text{log}\;n\right)df_{\lambda }. \end{array}$$
  Then the optimal tuning parameter λ* is chosen as the minimizer of *BIC*(λ):
  $$\begin{array}{c} \lambda^{*}={\text{argmin}}_{\lambda \in \Lambda }BIC\left(\lambda \right). \end{array}$$
2. *V*-fold cross validation (CV)
  The original dataset is first divided into *V* subsamples with an equal size, where *V* is a user-specified positive integer, such as *V* = 5. The *r*th subsample is taken as the testing set and the remaining (*V* − 1) subsamples are merged as the training set.
  Applying (11) to the training set gives us an estimator of *γ*, denoted *γ*^(−*r*)^(λ). Then we evaluate *ℓ*(*γ*) in (11) at *γ* = *γ*^(−*r*)^(λ) based on the *r*th testing set, and let *ℓ*^(*r*)^(*γ*^(−*r*)^(λ)) denote the resulting value. Finally, we compute
  $$\begin{array}{c} CV\left(\lambda \right)=\frac{1}{V}\sum_{r=1}^{V}\ell^{\left(r\right)}\left(\gamma^{\left(-r\right)}\left(\lambda \right)\right). \end{array}$$
  The optimal tuning parameter λ* is then determined by the minimizer of *CV*(λ):
  $$\begin{array}{c} \lambda^{*}={\text{argmin}}_{\lambda \in \Lambda }CV\left(\lambda \right). \end{array}$$
  This approach can be realized by employing the function cv.ncvreg in the R package ncvreg.

## 5 Syntax of R package

In this section, we present our developed R package, **AteMeVs**, which implements the estimation procedures described in Section 3, together with the details in Section 4. The developed R package utilizes the available R packages: **MASS** and **ncvreg**. The former is used to generate a multivariate normal distribution to address (10), and the latter is used to implement penalty functions as outlined in Section 4. Below, we describe the syntax of the developed functions that implement the step-by-step estimation procedure in Section 3.

### SIMEX_EST

The function SIMEX_EST implements Steps 1-3 in Section 3.1, given by

SIMEX_EST(data, PS = “logistic”, Psi = seq(0,1,length = 10), px = p, K = 200, extrapolate=“quadratic”, Sigma_e, replicate = “FALSE”, RM = rep(0,px)).

The arguments in this function include

- data: an *n* × (*p* + 2) matrix of a dataset. The first column records the observed outcome, the second column displays the values for the binary treatment, and the remaining columns store the observed measurements for the confounders.
- PS: a specification of a link function *g*(⋅) in (2). logistic refers to the logistic regression function (3), probit reflects the probit model (4), and cloglog gives the complementary log-log regression model (5).
- Psi: the specification of C in Step 1.
- px: the dimension of *X*.
- K: a positive integer *K* in Step 1.
- extrapolate: the extrapolation function in Step 3. quadratic reflects the quadratic polynomial function (16), linear gives the linear polynomial function (17), RL is the rational linear function (18), and cubic refers to the cubic polynomial function (19).
- Sigma_e: the covariance matrix Σ_*e*_ for the measurement error model (9).
- replicate: the identification of the availability of repeated measurements in the confounders. replicate = “FALSE” represents no repeated measurements and replicate = “TRUE” indicates that repeated measurements exist in the dataset. The default is set as replicate = “FALSE”.
- RM: a $p_{X}$ dimensional user-specified vector with each entry representing the number of repetitions for the respective confounder. For example, RM = c(2,2,3) indicates that three confounders in *X* have repeated measurements, where the first and second confounders have two repetitions and the third one has three repetitions. The default of RM is set as the $p_{X}$-dimensional zero vector, i.e., RM = rep(0,px).

In the argument data, the potential outcome can be continuous or binary and the treatment in the second column is designed to be binary. For the columns of confounders, one should place error-prone confounders from the third to the (px+2)-th column, and the remaining columns record precisely-measured confounders in *Z*. The argument PS is used to specify a link function *g*(⋅) that is used to characterize the propensity score in model (2), where the logistic regression model (3) is taken as the default. Two arguments Psi and K are used to generate the working data $X_{i}^{*}\left(k,\psi \right)$ in Step 1 in Section 3.1. The default of Psi is given by seq(0,1,length = 10), i.e., an interval [0, 1] with equal width divided into *M* = 10 subintervals, and the default of K is set as 200. px reflects the dimension of error-prone confounders, with the default value set as the dimension of all confounders *W*_*i*_, revealing that all confounders may be subject to measurement error. Setting px = 0 accommodates the situation where all confounders are precisely measured and there is no need to correct the measurement error effects. On the contrary, if confounders do involve measurement error, specifying px = 0 yields the *naive* estimate of *τ*_0_ which ignores the measurement error effects. The argument extrapolate contains commonly used working functions for extrapolation listed in Section 4.2. The default of the working extrapolation function is taken as the quadratic function.

Finally, Sigma_e records the covariance matrix Σ_*e*_, which can be user-specified or estimated by using auxiliary information, as described in Sections 3.2-3.4. Two arguments replicate and RM are used to indicate the availability of repeated measurements and the way of generating working data. Specifically, when replicate = “FALSE”, then (10) is implemented to generate the working variables in the main dataset for Cases 1, 2, and 4, respectively described in Sections 3.1, 3.2, and 3.4. On the other hand, the argument replicate = “TRUE” reflects Case 3 described in Section 3.3, which uses (14) to replace Step 1 in Section 3.1. The argument RM is in use to accompany with the argument replicate. When replicate = “FALSE”, no repeated measurements are available in the sample, and RM should be set as RM = rep(0,px). In contrast, if replicate = “TRUE”, there are repeated measurements for the confounders, and in this case, users should specify the number of repeated measurements for each confounder by setting a proper value for RM. For example, setting RM = c(2,2,3) represents that three confounders have repeated surrogate measurements, having 2, 2, and 3 replicates, respectively. When all arguments are specified, the output of this function gives a vector $\tilde{\gamma }$ as defined in Step 3.

### VSE_PS

The function VSE_PS, reflecting *variable selection and estimation of propensity scores*, is used to implement Step 4 in Section 3.1. The input function is given by

VSE_PS(V, y, method=“lasso”, cv=“TRUE“, alpha = 1),

with the following arguments:

- V: a (*p* + 1) × (*p* + 1) matrix *V*_*n*_ in (11).
- y: a (*p* + 1)-dimensional vector $\tilde{\gamma }$ in (11).
- method: it reflects the penalty function *ρ*_λ_(⋅) in (11) with choices presented in Section 4.3, where “lasso”, “scad” and “mcp” are given by (20), (21) and (22), respectively.
- cv: the method for choosing the tuning parameter λ. cv=“TRUE” suggests the use of the cross-validation method and cv=“FALSE” allows the use of the BIC, described in Section 4.4.
- alpha: a constant *α* ∈ [0, 1] in (23).

The argument V is a user-specified matrix, with the default set as the identity matrix, and y represents a vector derived by the output of SIMEX_EST. The argument method provides the penalty functions in Section 4.3 that are implemented in the R package **ncvreg**. The argument cv gives two choices to determine the optimal tuning parameter, respectively determined by cross-validation and BIC. Finally, alpha reflects a user-specified value *α* in (23), with the default value alpha = 1 that recovers the lasso method.

The output of this function gives a (*p* + 1)-dimensional vector of the estimator of *γ*. In this vector, components with zero values represent confounders that are unimportant and should be excluded; components with nonzero values identify important confounders entering the treatment model (12).

### EST_ATE

Upon the implementation of Steps 1-4, we then use the function EST_ATE to estimate ATE, as discussed in Step 5. The implementation is given by

EST_ATE(data, PS = “logistic”, Psi = seq(0,1,length = 10), K = 200, gamma, px = p, extrapolate=“quadratic”, Sigma_e, replicate = “FALSE”, RM = 0, bootstrap = 100).

All the arguments in this function are the same as those in SIMEX_EST, except for the argument gamma. The argument gamma records the estimate obtained from the implementation of Steps 1-4, and is used to estimate the propensity score ${\hat{\pi }}_{i}\left(k,\psi \right)$ in Step 5. The function EST_ATE provides the final estimate of ATE.

Furthermore, to provide a variance estimate and the resulting p-value for the estimated ATE, we employ the bootstrap algorithm by repeatedly running the proposed procedure to a sequence of bootstrap samples; then using the resulting estimates of ATE, we compute the sample variance of those estimates; taking this as a bootstrap variance for the initially obtained estimate of ATE, we calculate an associated p-value. The argument bootstrap is used to specify the number of bootstrap samples the user wishes to consider; its default value is set as 100. Function EST_ATE outputs values with headings estimate, variance, and p-value, which are a point estimate, the associated variance estimate, and the resulting p-value of *τ*_0_, respectively.

## 6 Numerical studies

### 6.1 Implementation of AteMeVs

In this section, we implement the R package **AteMeVs** to the METABRIC data described in Section 1. The dataset contains the information for 1422 patients, together with 331 gene expressions. Following the notation in Section 2, we define *Y* and *T* as “overall_survival” and “hormone_therapy”, respectively. We let *W* denote those gene expressions. The following code is used to prepare for the dataset.


read.table(“C://METABRIC_causal.csv”, sep = “,”, header = TRUE) ->
data_METABRIC

library(MASS)
library(ncvreg)
library(AteMeVs)

data = data_METABRIC[, 1:280]
gene = colnames(data_METABRIC)[3:280]
set.seed(20651252)
n = dim(data)[1]
p = dim(data)[2] - 2


We now demonstrate Steps 1-3 by using the function SIMEX_EST and by setting *K* = 10 and C to include the cutpoints equally dividing the interval [0, 2] into *M* = 10 subintervals. Since there is no additional information to estimate Σ_*e*_, we follow Case 1 in Section 2.3 to specify Σ_*e*_ as a diagonal matrix with common value s2 = 0.2. As noted in [32], measurements of gene expressions are subject to measurement error, and therefore, we specify px = p, which is 331. The implementation is given below:


Psi = seq(0, 2, length = 10)
K = 10
s2 = 0.2

y =  as.vector(SIMEX_EST(data, Psi, K, px = p, Sigma_e = diag(s2, p)))
matrix(y,ncol=2)
               [,1]         [,2]
  [1,] -0.132002775 -0.098403692
  [2,]  0.192014131 -0.247497662
  [3,]  0.333889793  0.153789525
  [4,] -0.470314553 -0.486142020
  [5,] -0.164594040 -0.040468911
  [6,] -0.190031631  0.041317059
  [7,]  0.443131214  0.108216814
  [8,] -0.612154496  0.014734219
  [9,]  0.201441389 -0.012663900
 [10,]  0.611405760 -0.242673669


Due to the space constraint, we report partial results for the output y above to show the estimate $\tilde{\gamma }$.

Next, we use $\tilde{\gamma }$ to demonstrate variable selection in Step 4. Since *V*_*n*_ in (11) is user-specified, we follow [23] and set *V*_*n*_ as the identity matrix. To see the impact of variable selection by different methods, we examine three penalty functions (20), (21), and (22). Detailed demonstrations with an application of the function VSE_PS are given below. We also display some results in the command “VS” as follows.


V = diag(1, length(y), length(y))
est_lasso_cv = VSE_PS(V, y, method = “lasso”, cv = “TRUE”)
est_scad_cv = VSE_PS(V, y, method = “scad”, cv = “TRUE”)
est_mcp_cv = VSE_PS(V, y, method = “mcp”, cv = “TRUE”)
cbind(est_lasso_cv,
      est_scad_cv,
      est_mcp_cv) -> VS
rownames(VS) = gene
VS
         est_lasso_cv est_scad_cv est_mcp_cv
brca1       0.0000000   0.0000000  0.0000000
brca2       0.0000000   0.0000000  0.0000000
palb2       0.0000000   0.0000000  0.0000000
pten        0.0000000   0.5182603  0.5230828
tp53        0.0000000   0.0000000  0.0000000
atm         0.0000000   0.0000000  0.0000000
cdh1        0.0000000   0.0000000  0.0000000
chek2       0.5457605   0.6601017  0.6649249
nbn         0.0000000   0.0000000  0.0000000
nf1        -0.5277423  -0.5634578 -0.5586342
stk11       0.0000000   0.0000000  0.0000000
bard1       0.0000000   0.0000000  0.0000000
mlh1        0.0000000   0.0000000  0.0000000
msh2        0.5342537   0.6485958  0.6534201
msh6        0.0000000   0.0000000  0.0000000
pms2        0.0000000   0.0000000  0.0000000
epcam       0.0000000   0.0000000  0.0000000
rad51c      0.0000000   0.0000000  0.0000000
rad51d      0.0000000   0.0000000  0.0000000
rad50       0.0000000   0.0000000  0.0000000
rb1        -0.8071897  -0.8429033 -0.8380785
rbl1        0.0000000   0.0000000  0.0000000
rbl2        0.0000000   0.0000000  0.0000000
ccna1       0.0000000   0.0000000  0.0000000
ccnb1       0.0000000   0.0000000  0.0000000
cdk1        0.0000000   0.0000000  0.0000000
ccne1       0.0000000   0.0000000  0.0000000
cdk2        0.0000000   0.0000000  0.0000000
cdc25a     -0.5527873  -0.5885001 -0.5836741
ccnd1       0.5691736   0.6835180  0.6883442
cdk4        0.5165555   0.6309001  0.6357266
cdk6       -0.7121596  -0.7478720 -0.7430453
ccnd2       0.0000000   0.0000000  0.0000000
cdkn2a     -0.5822496  -0.6179617 -0.6131343
cdkn2b      0.0000000   0.0000000  0.0000000
myc        -0.6168412  -0.6525533 -0.6477256
cdkn1a      0.0000000   0.0000000  0.0000000
cdkn1b      0.0000000   0.0000000  0.0000000
e2f1        0.0000000   0.0000000  0.0000000
e2f2        0.0000000  -0.5323233 -0.5274951


Variable selection shows that zero values correspond to unimportant gene expressions and nonzero ones suggest important gene expressions. The results show that informative gene expressions are sparse regardless of variable selection methods. In the partial results displayed here, we observe that some gene expressions, such as “e2f2” and “pten”, are selected by (21) and (22) but not by (20). Moreover, selected gene expressions contain “cdk4”, “cdk6”, and “ccnd1”, which is consistent with the findings of [38, 39]; they found that “ccnd1” was associated with a good breast cancer prognosis and cdk6 has been shown to be regulated and influenced by several mitogenic signaling pathways in breast cancer.

Finally, using the selected gene expressions, we estimate ATE by using the function EST_ATE; the estimation result is shown as follows:


ate_lasso_cv =  EST_ATE(data,
                        gamma = est_lasso_cv,
                        px = p,
                        Sigma_e = diag(s2, px))
ate_scad_cv =  EST_ATE(data,
                       gamma = est_scad_cv,
                       px = p,
                       Sigma_e = diag(s2, px))
ate_mcp_cv =  EST_ATE(data,
                      gamma = est_mcp_cv,
                      px = p,
                      Sigma_e = diag(s2, px))
> ate_mcp_cv
     estimator   variance      p-value
[1,]    1.4773 0.04885097 2.326125e-11
> ate_scad_cv
     estimator   variance      p-value
[1,]  1.631049 0.04049768 5.275382e-16
> ate_lasso_cv
     estimator  variance   p-value
[1,]  1.116406 0.0357837 3.597e-09


An estimate of ATE is 1.4773, 1.631049, and 1.116406, respectively corresponding to the estimates of *γ* derived from (20), (21), and (22); and associated variance estimates derived by the three methods are 0.04885097, 0.04049768, and 0.0357837, respectively. All the three resulting p-values are smaller than the significance level 0.05, suggesting that the chemotherapy treatment has a positive causal effect on increasing the survival of a patient. These results are derived by accommodating the effects of informative gene expressions, including “ccnd1”, “cdk4”, and “cdk6”, which are also identified to be informative by [9].

### 6.2 Comparisons of AteMeVs with other methods

To highlight the advantages of the package **AteMeVs** and underscore the importance of addressing issues of measurement error and variable selection, we consider two additional scenarios: (i) using **AteMeVs** without implementing VSE_PS, and (ii) using existing packages **iWeigReg** and **CausalGAM**, in comparison to the use of **AteMeVs** with different penalty functions. In Scenario (i), we correct for measurement error in confounders but do not address the exclusion of irrelevant confounders; in Scenario (ii), we aim to estimate ATE without taking measurement error and variable selection into account. We summarize the numerical results in Table 1, where ‘EST’ represents the estimate of ATE, ‘VAR’ is the variance associated with the estimated ATE derived from the packages, and ‘p-value’ is the p-value derived from testing the null hypothesis *H*_0_: *τ*_0_ = 0.

**Table 1:** Comparisons of estimation methods. LASSO(*x*) is the usage of the package AteMeVs with the LASSO penalty function, SCAD(*x*) is the usage of the package AteMeVs with the SCAD penalty function, MCP(*x*) is the usage of the package AteMeVs with the MCP penalty function, Full(*x*) refers to Scenario (i), where *x* = 0.2, 0.5 or 0.7, representing identical diagonal elements in Σ_*e*_. The usage of iWeigReg and CausalGAM refer to Scenario (ii).

	Method	Estimator of ATE		
		EST	VAR	p-value
**AteMeVs**	LASSO(0.2)	1.477	0.049	2.326e-11
	LASSO(0.5)	1.739	0.043	6.83e-17
	LASSO(0.7)	1.590	0.071	2.525e-09
	SCAD(0.2)	1.631	0.040	5.275e-16
	SCAD(0.5)	1.115	0.102	0.000
	SCAD(0.7)	0.661	0.097	0.034
	MCP(0.2)	1.116	0.036	3.597e-19
	MCP(0.5)	1.274	0.066	7.084e-07
	MCP(0.7)	1.440	0.099	4.599e-06
Scenario (i)	Full(0.2)	0.027	0.031	0.878
	Full(0.5)	0.300	0.079	0.285
	Full(0.7)	0.005	0.075	0.986
Scenario (ii)	**iWeigReg**	3.168	0.741	0.000
	**CausalGAM**	0.121	0.038	0.534

The results show that the package **AteMeVs** performs stably, irrespective to the degree of measurement error and the choice of the penalty function; significant causal effects are revealed by the use of **AteMeVs**. In contrast, Scenario (i) shows insignificant causal effects with p-values greater than 0.05, which might be caused by the involvement of irrelevant confounders, even though measurement error correction is taken into account. Moreover, with the ignorance of measurement error effects and variable selection, it is interesting that the package **iWeigReg** shows evidence for the significance of the causal effects, but the EST and VAR are greater than those derived by the package **AteMeVs**. On the other hand, **CausalGAM** does not provide evidence for suggesting ATE differs zero.

## 7 Discussion

The inverse-probability-weighting estimation method and its variants have proved to be useful for estimating the average treatment effect within the causal inference framework. However, their applications are hindered by two critical conditions. The validity of those methods relies on the proper determination of propensity scores and the use of the precise measurements of the covariates. When data lack these features [23], introduced a simulation-based method that adapts the inverse-probability-weighting scheme to accommodate measurement error effects as well as variable selection for calculating propensity scores. In this paper, we develop an R package, called **AteMeVs**, to extend the method proposed by [23]. This package provides analysts a user-friendly tool for estimating the average treatment effect when working with error-contaminated data and inconsequential confounders.

As the package **AteMeVs** is designed to handle classical measurement error model (9), biased estimation is anticipated when model (9) is not feasible; the impact of the violation of the model (9) was explored by [40] for survival analysis with covariate measurement error. Further, the development of the package **AteMeVs** lies on the correct parametric modelling for the propensity score. When such an assumption is untrue, estimation results obtained from using **AteMeVs** may become invalid. However, in applications, the relationship between the treatment and the confounders can be complex, making it difficult to have straightforward representation through a convenient parametric model. It is useful to introduce semiparametric models to characterize propensity scores.

While the package **AteMeVs** offers flexibility in handling measurement error and variable selection, it has limitations. Currently, the package focuses on continuous error-prone random variables, and it cannot handle error-contaminated confounders that are mixed with both continuous and discrete variables. Another notable issue concerns the size of data. The **AteMeVs** is basically developed for settings where the number of confounders is smaller than the sample size, which is driven by the setup considered in [23]. It is interesting to generalize the method in [23] to handle high-dimensional error-prone data, where the dimension of confounders can be diverging as the sample size approaches infinity. Creating R packages to conduct causal inference about such data can be useful.

Finally, the package **AteMeVs** developed here can only handle outcomes with complete observations. In the presence of incomplete responses with error-contaminated covariates, such as survival data with covariate measurement error, it is important to address both the censoring effects (e.g., [41]) and the measurement error effects when estimating causal effects. It is interesting to devise causal inference methods to handle such data and then develop R packages accordingly to extend the application scope of the package **AteMeVs**.
